# Supplementary material for: Age Rather Than Supplementation with Oat β-Glucan Influences Development of the Intestinal Microbiota and SCFA Concentrations in Suckling Piglets
Source: Animals (Basel). 2023 Apr 14;13(8):1349. doi: 10.3390/ani13081349 (PMC10135274; doi:10.3390/ani13081349)
Supplement: Supplementary file 1 [file animals-13-01349-s001.zip › Arapovic_et_al_Supplementary Figure S1-S3.pdf]

SUPPLEMENTARY FIGURES

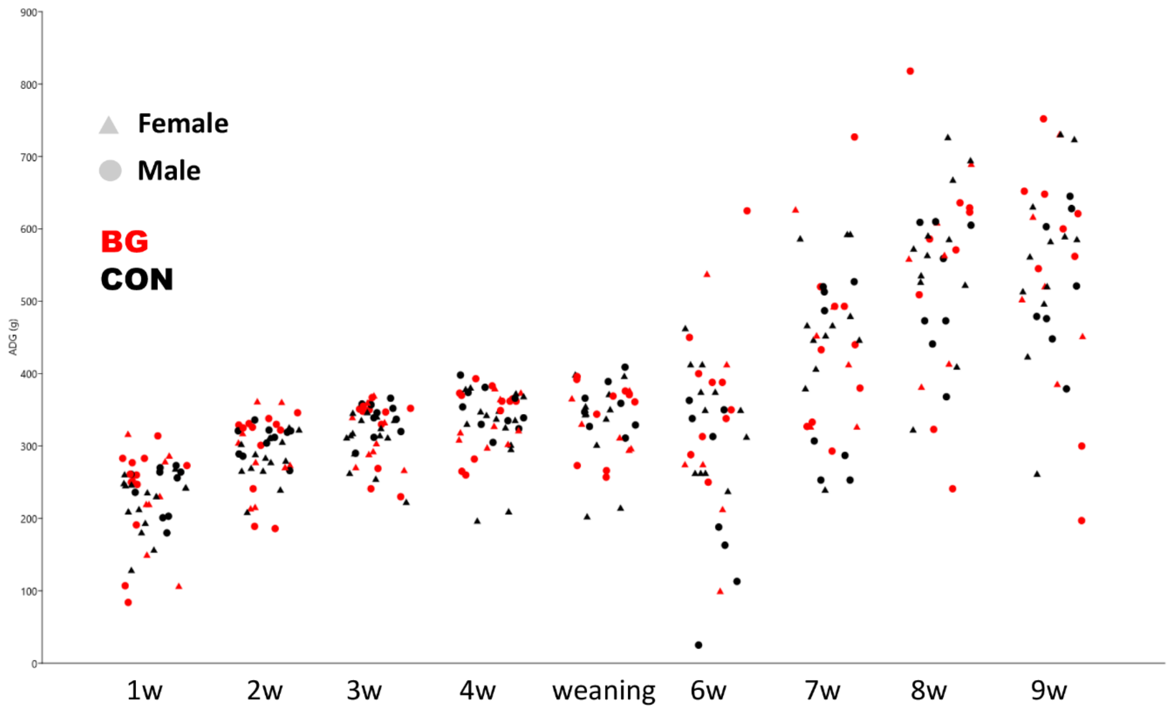

Supplementary figure S1. Data on average daily weight gain of the pigs included in the study. Average daily weight gain (ADG) for piglets included in the study from birth to nine weeks of age (±SE). ADG from week 1 – weaning was related to the birth weight, whereas the ADG from 6w - 9w age was related to the weight at weaning. BG=β-glucan supplemented group, CON=control group.

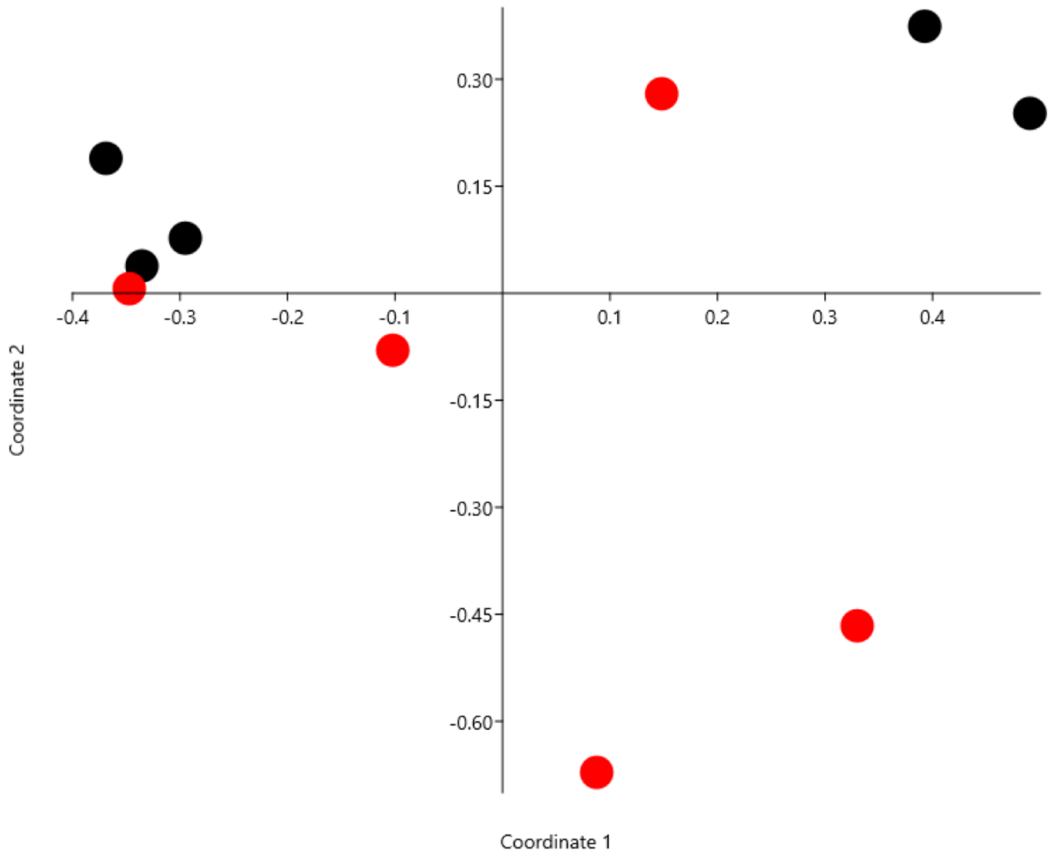

Supplementary figure S2. PCoA on colonic digesta from euthanized piglets

Principal coordinates analysis (PCoA) based on Bray Curtis distances on colonic digesta from euthanized piglets did not reveal a separate clustering of samples between control (black) and β-glucan (red) supplemented piglets.

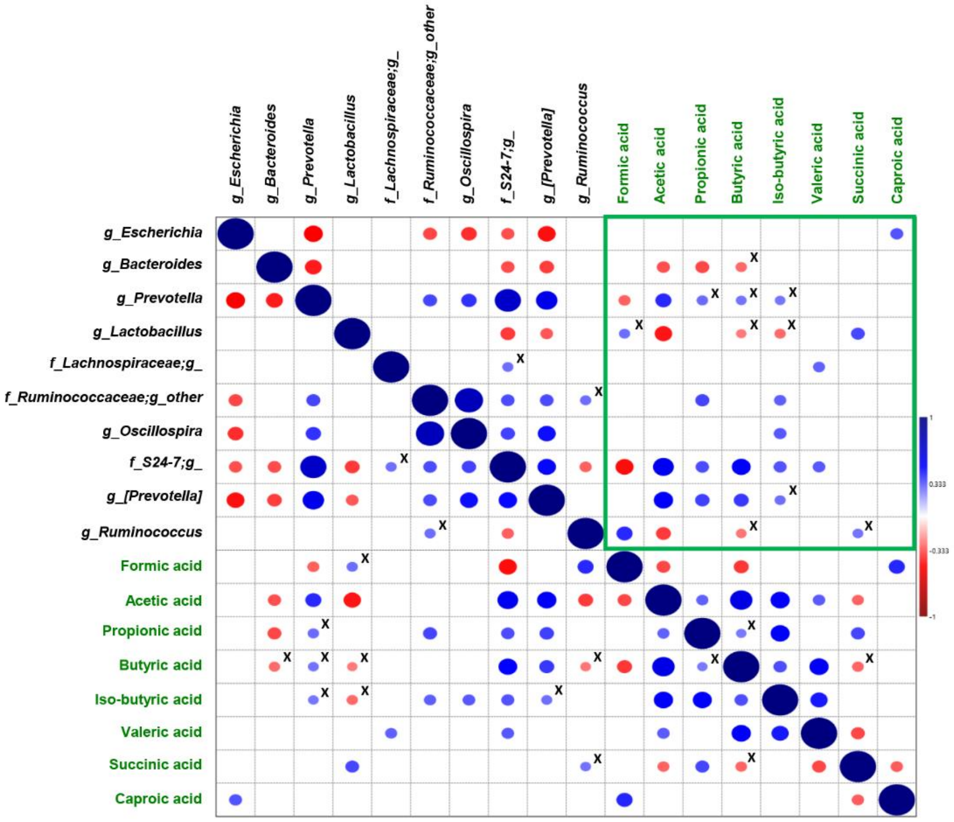

Supplementary figure S3. **The correlation between the ten top genera and SCFA**

The correlation between faecal bacteria from swab samples at genus level (the top 10 genera) and the plasma concentration of short-chain fatty acids and caprionic acid. All pairwise correlations indicated with a dot, represents significant correlations. Correlations marked with an "x" was however not significant after False Discovery Rates (FDR) adjustments. Positive correlations are shown in blue whereas negative correlations in red. The size of the dots represents the strength of the correlation (larger dots represent stronger correlations).
