# Supplementary material for: Age Rather Than Supplementation with Oat β-Glucan Influences Development of the Intestinal Microbiota and SCFA Concentrations in Suckling Piglets
Source: Animals (Basel). 2023 Apr 14;13(8):1349. doi: 10.3390/ani13081349 (PMC10135274; doi:10.3390/ani13081349)
Supplement: Supplementary file 1 [file animals-13-01349-s001.zip › Arapovic_et_al_Supplementary Table S1-S4.pdf]

SUPPLEMENTARY TABLES

Supplementary table S1. Dietary content of creep feed for suckling piglets.

Content	Amount
NE,MJ	10
Energy (MJ/kg)	13.3
Water %	12
Protein (g/kg)	151
Fat (g/kg)	69
Crude fibre (g/kg)	44
Ash (g/kg)	52
Sodium (g/kg)	3
Potassium (g/kg)	8
Calcium (g/kg)	4.4
Lysine (g/kg)	10.7
Metione (g/kg)	3.9
Vitamin A (IE/kg)	5000
Vitamin D3 (IE/kg)	500
Vitamin E (IE/kg)	120
Selenium (mg/kg)	0.5
Phosphorus (g/kg)	5
Nitrogen (g/kg)	24
Soluble fibre(g/kg)	110
Insoluble fibre(g/kg)	150

Supplementary table S2. The excluded outliers

The excluded outliers lager than 2.5 units, bacteria

**Bacteria**	**Outliers**
Actinobacteria	1
Bacteroidetes	2
Fusobacteria	2
Escherichia	1
Bacteroides	2
Prevotella	2
Lactobacillus	2
S24_7:g	2
[*Prevotella*]	1

Supplementary table S3. SCFA and caproic acid in colon digesta from euthanized piglets

Short chain fatty acids and caproic acid concentration in colon digesta collected from euthanized piglets fed with β-glucan supplement (BG) and the control group (CON) at weaning. (Average ± SE)

	Colon digesta BG		Colon digesta CON	
**SCFA(µM)**	Average	SE	Average	SE
Formic acid	0,68	0,10	0,42	0,12
Acetic acid	24,1	4,73	29,2	3,90
Propionic acid	8,27	2,31	12,6	2,08
Butyric acid	3,41	1,30	7,30	1,51
Isobutyric acid	0,84	0,18	0,97	0,18
Succinic acid	0,58	0,16	0,61	0,09
Valeric acid	1,24	0,37	1,97	0,33
Isovaleric acid	0,63	0,15	0,80	0,15
Caproic acid	0,24	0,10	0,36	0,11

SE= Standard error, BG=Beta glucan supplemented group, CON=control group

Supplementary table S4. SCFA and caproic acid in plasma from euthanized piglets

Short chain fatty acids and caproic acid concentration in plasma collected from euthanized piglets fed with β-glucan supplement (BG) and the control group (CON) at weaning. (Average ± SE)

	Jugular vein BG		Jugular vein CON		Portal vein BG		Portal vein CON	
**SCFA(μM)**	Avg.	SE	Avg.	SE	Avg.	SE	Avg.	SE
Formic acid	234	70,8	274	76,4	431	59,1	341	86,7
Acetic acid	9,83	1,99	30,4	13,0	70,6	15,4	102	39,0
Propionic acid	0,62	0,14	1,78	0,79	48,5	14,0	65,4	22,9
Butyric acid	0,51	0,12	1,36	0,63	20,9	9,30	25,5	12,5
Isobutyric acid	0,64	0,04	1,01	0,22	4,38	1,37	7,42	2,14
Succinic acid	6,43	0,92	8,65	2,99	20,6	7,04	21,47	5,42
Valeric acid	0,11	0,02	0,28	0,09	7,67	2,94	9,37	3,29
Isovaleric acid	0,71	0,03	0,88	0,09	3,47	0,81	5,49	1,56
Caproic acid	0,25	0,03	0,30	0,04	2,39	0,40	2,24	1,24

SE= Standard error, AVG=Average, BG=Beta glucan supplemented group, CON=control group
